# The Impact of Testosterone Supplementation on Surgical Complications in Gender-Affirming Mastectomy: A Meta-Analysis

**DOI:** 10.1007/s00266-026-05692-8

**Published:** 2026-02-24

**Authors:** Diwakar Phuyal, Sara Yacoub, Zoe E. Belardo, Antonio Rampazzo, Bahar Bassiri Gharb

**Affiliations:** Department of Plastic and Reconstructive Surgery, Cleveland Clinic Foundation, 9500 Euclid Ave, Desk A6-522, Cleveland, OH 44195 USA

**Keywords:** Gender affirming mastectomy, Gender affirming surgery, Top surgery, Testosterone supplementation

## Abstract

**Background:**

Patients seeking gender-affirming surgery are usually on hormone supplementation. We hypothesized that perioperative testosterone therapy increases hematoma following chest masculinizing surgery.

**Methods:**

Meta-analysis was conducted according to Preferred Reporting Items for Systematic Reviews and Meta-Analyses (PRISMA) guidelines. Studies with perioperative hormone status and surgical outcomes were included. Patient characteristics, testosterone use, surgical details, and outcomes were extracted. Meta-analysis and pooled analysis using aggregated patient-level data were conducted.

**Results:**

Fifteen studies were included, comprising 3,036 testosterone-treatment (TT) and 726 non-testosterone-treatment (NTT) patients. Overall complication rates were similar (11.08% vs. 9.80%; *p* = 0.29). Meta-analysis showed significantly higher hematoma in TT (OR 1.99, [1.19–3.34], *p* < 0.001). Other complications showed no significant differences: infection (OR 0.33 [0.07–1.59]), wound dehiscence (OR 0.68 [0.04–11.06]), seroma (OR 0.5 [0.15–1.57]), and venous thromboembolism (OR 0.20 [0.0–10.42]). Hematoma risk did not differ between patients who continued versus stopped testosterone (TT-C 8.73% vs. TT-S 7.80%; *p* = 0.54), or by cessation timing (*p* = 0.60). Mixed-effects meta-regression found incision type was not a significant moderator of hematoma (QM (1) = 0.34, *p* = 0.56) among TT and NTT. Without stratifying by testosterone, hematoma rates were higher with limited incisions than extended incisions (11.96% vs. 6.5%; *p* < 0.01).

**Conclusions:**

Testosterone therapy was associated with higher postoperative hematoma rates, while perioperative cessation did not reduce hematoma or overall complications. These findings support heightened perioperative awareness and vigilance for hematoma risk in patients receiving testosterone, while decisions regarding the timing of hormone therapy initiation should be carefully weighed against established mental health benefits.

**Level of Evidence III:**

This journal requires that authors assign a level of evidence to each article. For a full description of these Evidence-Based Medicine ratings, please refer to the Table of Contents or the online Instructions to Authors www.springer.com/00266.

## Introduction

Breast surgery is one of the most commonly performed procedures among gender affirming procedures, with a reported prevalence as high as 56.6% [1]. While the majority of the patients undergoing gender-affirming mastectomy are on hormone therapy, WPATH guidelines do not mandate testosterone therapy, as some patients, including nonbinary individuals, seek only the achievement of an androgynous flat chest [2, 3]. Some studies in cisgender populations have shown that testosterone supplementation increases the risk of venous thromboembolism (VTE), postoperative bleeding, hematoma, cardiovascular events, and mortality [3–9]. Yet, these findings are not consistent across all studies [4–6, 10]. This inconsistency has led to a lack of consensus in the literature regarding preoperative testosterone cessation and its potential impact on postoperative complications [10]. Moreover, research directly correlating gender affirming hormone therapy (GAHT) with postoperative outcomes remains limited. There are no evidence-based guidelines on whether to continue or temporarily cease GAHT, including testosterone, before surgery. Consequently, surgeons must weigh the potential surgical risks against the emotional and quality-of-life-associated detriments to their patients when deciding on hormone management in the perioperative period.

Given the increasing demand for gender-affirming care and surgery, this review aims to consolidate current data on surgical risks associated with preoperative and perioperative testosterone use in gender-affirming surgery (GAS). Specifically, we evaluated the impact of testosterone therapy on postoperative complications following gender-affirming mastectomy, with postoperative hematoma as the primary outcome and other surgical complications as secondary outcomes. We hypothesized that patients receiving preoperative testosterone therapy would have a higher risk of postoperative hematoma compared with those not receiving testosterone therapy.

## Methods

A comprehensive literature search was conducted in accordance with the Preferred Reporting Items for Systematic Reviews and Meta-Analyses (PRISMA) guidelines [11]. The databases Ovid Embase, Ovid Medline, and Cochrane Central were searched on September 30, 2024. The search strategy incorporated truncation, adjacency, Boolean operators, and indexing terms. A manual review of references was also performed. Studies on chest masculinizing surgery that reported data on both surgical outcomes and preoperative hormone status were included. Both retrospective and prospective studies were eligible. Studies were excluded if 1. surgical complications were not reported, 2. no reference to hormone therapy was made, 3. they were case reports, or 4. the article was not written in English. No studies were excluded based on duration of follow-up.

**Fig. 1:**
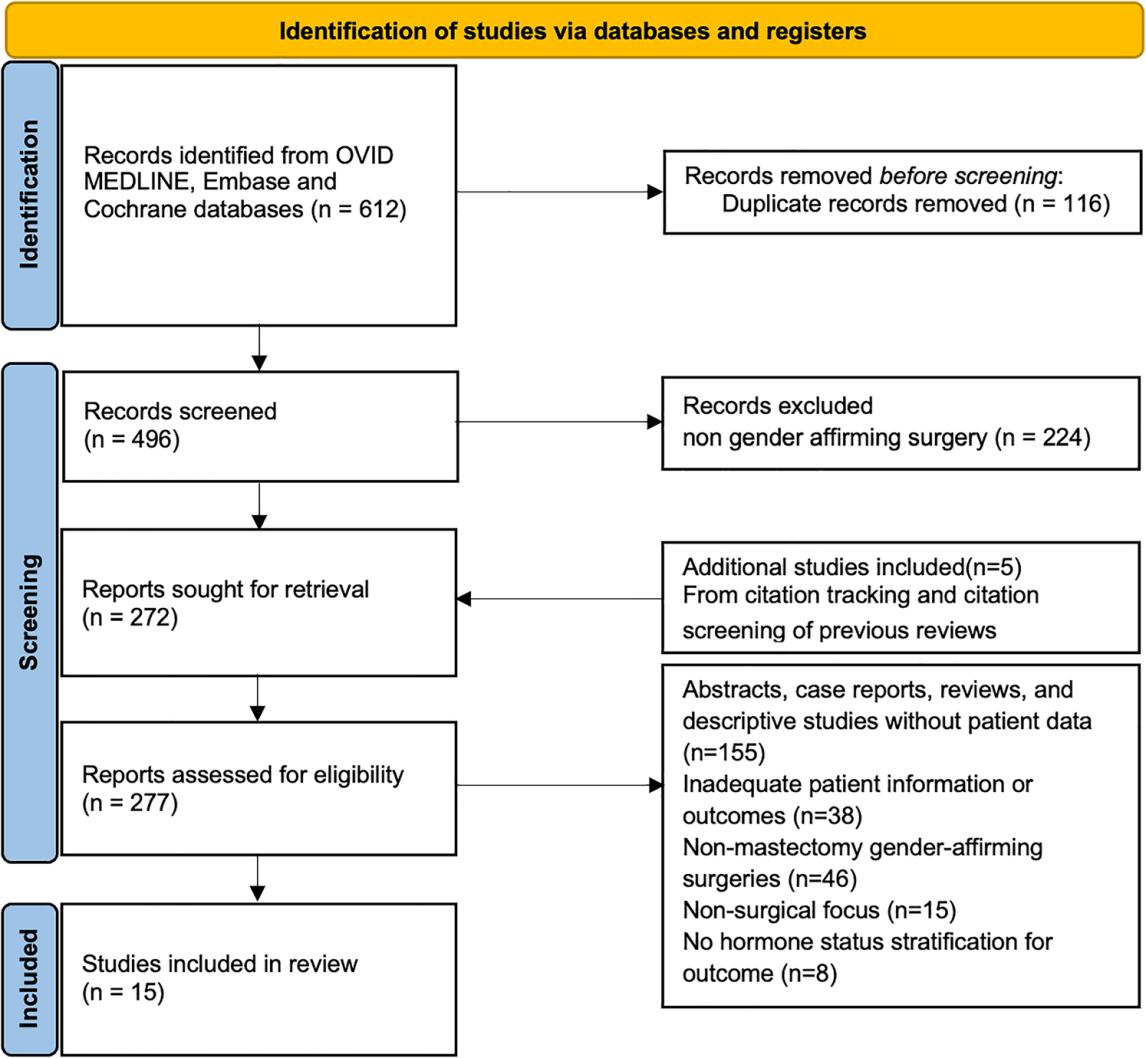
Flow diagram showing the study selection process for the systematic review

Three studies with overlapping patient populations treated by a single surgeon were identified; only one of these met the inclusion criteria and was included [12–14]. Four hundred and ninety-six articles were uploaded in Covidence (Veritas Health Innovation, Melbourne, Victoria, Australia). Title and abstract screening were conducted independently by two authors (DP and SY), and 277 articles were deemed eligible for full-text review (Fig. 1).

Three reviewers (DP, SY, ZB) independently reviewed full texts and extracted data using a predefined Microsoft Excel® (Version 16.77.1) based extraction form with iterative refinement during full-text review. Ultimately, 15 articles met the inclusion criteria and were used for data extraction.

The following data were extracted from each eligible article: level of evidence, size of study population, age, gender identity, body mass index (BMI), nicotine use, hormone dose and route of administration (if available), perioperative hormone use, complications, and secondary procedures.

Data collected from the included studies were systematically reviewed and quantitatively synthesized to assess surgical outcomes. Patients were classified as receiving testosterone therapy (TT) or not receiving testosterone therapy (NTT) based on reported hormone status in each study. The TT group included individuals on preoperative hormone therapy, whereas NTT includes only patients with no prior history of testosterone use or testosterone-naïve patients. Within the TT group, patients were further subdivided into those who continued testosterone therapy perioperatively (TT-C) and those who discontinued therapy before surgery (TT-S). Cessation protocols were analyzed according to each study’s reported timeline, and subgroup comparisons were performed for cessation intervals of 2, 3, 4, and 6 weeks prior to surgery to assess potential timing-related effects to explore potential acute effects of testosterone withdrawal versus continuation in the perioperative setting.

For this analysis, ‘acute complications’ were defined as events occurring in the immediate postoperative period and commonly reported across included studies, including hematoma, seroma, wound dehiscence, nipple–areolar complex (NAC) necrosis, and infection. Total hematoma was defined as any postoperative hematoma reported by the included studies, regardless of whether it required intervention. Hematoma requiring intervention was defined as hematomas necessitating surgical evacuation, aspiration, or procedural management, as specified by individual study definitions. Long-term or aesthetic complications (e.g., dog-ear deformity, hypertrophic scar formation, and revision surgery) were excluded due to inconsistent reporting and their delayed onset. Additionally, complications were compared between patients TT-C and TT-S.

A meta-analysis was conducted to evaluate the proportion of all complications, including total hematoma, hematoma requiring intervention, seroma, wound infection, wound dehiscence, VTE, and NAC necrosis, using studies that included both the TT and NTT groups. The meta-analysis was performed with random-effects models in R (metafor package) for comparisons between TT and NTT. For comparisons between the TT-C and TT-S groups, data were synthesized using pooled analyses of aggregated patient-level data due to the limited number of available studies. To account for variability in operative technique, hematoma rates were also stratified by surgical approach, comparing the extended incision and limited incision techniques. A mixed-effects meta-regression was conducted as an exploratory analysis to assess whether surgical type moderated the association between TT and NTT. Furthermore, a pooled analysis was performed to evaluate the main effects of surgical technique without testing for moderation by testosterone status. “Extended incision” techniques included double-incision, buttonhole, and similar approaches involving wide exposure with pedicled or free NAC grafting. “Limited incision” techniques included periareolar, circumareolar, and keyhole approaches, characterized by pedicled nipple preservation and more limited exposure. To evaluate potential confounding by nicotine use, a study-level sensitivity analysis excluding studies reporting nicotine use in both TT and NTT groups was performed, as individual patient-level smoking data were unavailable. NAC necrosis data were stratified based on the use of pedicled NAC preservation versus free NAC grafting.

For pooled analysis using aggregated patient-level data, categorical variables were compared using chi-square tests when expected cell count assumptions were met (expected counts ≥5); Fisher’s exact test was used otherwise. Continuous variables were compared using Welch’s t-test to account for unequal variances. A Cochran–Armitage test was utilized to evaluate trends across ordered cessation intervals. Heterogeneity in the meta-analyses was assessed using the I^2^ statistic, with values of 0–25% indicating low heterogeneity, 26–50% moderate heterogeneity, and >50% substantial heterogeneity. A two-sided *p*-value < 0.05 was considered statistically significant. No formal adjustment for multiple comparisons was performed, as analyses were hypothesis-driven with a predefined primary outcome.

The quality of the included studies was evaluated by two authors (DP and ZB) using the American Society of Plastic Surgeons’ Rating Levels of Evidence and Grading Recommendations. Discrepancies were resolved through review by a third author (SY). All statistical analyses were performed using RStudio (Version 2023.12.1+402) and IBM® SPSS® Statistics (Version 27).

## Results

Three thousand seven hundred sixty-two patients from 15 articles were included for data analysis, among whom 3036 were on testosterone preoperatively and 726 were not. Gender orientation was explicitly specified for 987 patients across six articles (Table 1). Of these, 960 were transgender men, 21 identified as nonbinary, and 6 were categorized as ‘other’. However, for the remaining 2981 patients undergoing gender-affirming mastectomy, their specific gender orientation was not clearly identified. The mean age of patients in the TT group was 26.10 ± 7.81 years, compared to 26.82 ± 6.38 years in the NTT group (*p* = 0.19). The mean BMI was 25.88 ± 5.16 kg/m^2^ in the TT group and 25.31 ± 5.51 kg/m^2^ in the NTT group (*p* = 0.33). Nicotine use was reported in 17.90% of the TT group compared to 25.70% in the NTT group (*p* = 0.029).

**Table 1:** Characteristics of all included studies: patient demographics, outcomes, outcomes by hormone use, and follow-up time

S.N.	Author, year	Level of evidence	Number of patients	Age (yrs)	BMI ± SD (kg/m^2^)	Nicotine Use	Comorbidities	Gender identification
1	Cregten-Escobar et al.,2012	IV	202	31.0 ± 10.0	25 ± 4	NR	NR	NR
2	Berry et al.,2012	III	100	Median age: 28.0 (IQR NR)	NR	29 (29.0)	Asthma: 12 (12.0) Hypertension: 1 (1.0) Hypothyroidism: 1 (1.0)	NR
3	McEvenue et al.,2018	III	679	26.3 ± 9.0	26.2 ± 5.4	NR	NR	NR
4	Top and Balta,2017	IV	52	29.1 ± 8.9	23.4 ± 3.8	NR	NR	NR
5	Donato et al.,2017	III	130	28.0 ± 9.0	28.3 (SD NR)	18 (13.8)	Diabetes: 2 (2.0) Asthma: 16 (12.0) Obstructive sleep apnea: 5 (4.0)	NR
6	Frederick et al.,2017	III	88	Median age: 24.0 (IQR NR)	22.3 (SD NR)	NR	NR	88 transgender men
7	Watanabe et al.,2019	IV	152	27.4 ± 6.1	22.7 ± 3.1	NR	Asthma: 3 (2.0)	NR
8	Ederer et al., 2021	IV	180	28.0 ± 9.5	26.7 ± 5.9	67 (37.2)	Coagulation disorder: (5.0)	180 transgender men
9	Rothenberg et al,2021	III	948	29.1 ± 9.5	27.2 ± 5.8	323 (34.1)	NR	NR
10	Wu et al.,2022	IV	236	25.0 ± 8	29.5 ± 6.6	NR	Diabetes: 6 (2.5) Thrombophilia: 1 (0.4)	236 transgender men
11	Tang et al.,2022	III	209	Median age at referral: 16.0 (IQR 2)	NR	8 (3.8)	NR	182 transgender men 21 nonbinary individuals 6 other
12	Robinson et al. ,2023	III	490	26.0 ± 8.6	25.7 ± 5.4	49 (10.0)	Diabetes: 8 (1.6) Coagulopathy: 7 (1.4) Cardiovascular disease: 21 (4.3)	NR
13	Rysin et al.,2023	III	170	21.6 ± 6.3	NR	NR	Diabetes: 1	170 transgender men
14	Huber et al.,2024	IV	104	26.0 ± 6.7	26.2 ± 4.6	NR	NR	104 transgender men
15	Tamulevicius et al.,2024	IV	22	23.6 ± 7.2	25.9 ± 5.1	NR	NR	NR

S.N.	Type of surgery	Perioperative hormone use	Hematoma (%)	Seroma (%)	NAC Graft Failure (%)	Wound dehiscence/infection (%)	Others	Follow-up time
1	Periareolar: 62	TT-S: 202 (Suspended 6 weeks preoperatively)	TT-S: 20 (9.9)	NR	NR	NR	Total reoperation: 141 breasts	NR
	DIPN: 65							
	DIFNG: 75							
2	Periareolar: 15	TT: 88 NTT: 12	TT: 6 (6.8) NTT: 0	NR	Partial NAC loss: 1 (1.0) Total NAC loss: 1 (1.0) *	3(3.0) *	NR	7 months
	DIFNG: 85							
3	Periareolar: 104	TT: 463 NTT: 216 Perioperative use: NR	Minor hematoma: 33 (4.9) Major hematoma: 11 (1.6) *	44 (6.5) *	Partial NAC loss: 3 (0.4)	28 (4.1) *	Need for surgical intervention: 11 (1.6) *	5.5 months
	DIFNG: 575							
4	Periareolar: 24	TT-S: 30 (Suspended 4 weeks preoperatively) NTT: 22	TT-S: 2 (3.8) NTT: 0	0	NTT: 5 (22.7)	0	Need for surgical intervention: 1 (2.0) *	28 months
	Vertical: 12							
	Apron flap technique: 16							
5	Periareolar: 20	TT-C: 106 NTT: 24	TT-C: 17 (16.0) NTT: 1 (3.4)	9 (6.9) *	0	NR	Surgical intervention: 12 (9.2) *	6 months
	DIFNG: 110							
6	Periareolar: 40	TT: 65 NTT: 23 Perioperative use: NR	TT: 8 (12.3) NTT: 0	0	0	0	NR	12 months
	DIFNG: 48							
7	Periareolar: 148	TT: 133 NTT: 19 Perioperative use: NR	TT: 14 (10.5) NTT: 1 (5.3)	NR	NR	NR	NR	NR
	DIFNG: 4							
8	Periareolar: 28	TT-C: 180	TT-C: 22 (12.2)	TT-C: 2 (1.1)	TT-C: 5 (2.8)	TT-C: 2 (1.1)	TT-C: Wound healing disturbance: 4 (2.2)	6.3 months
	DIFNG: 133							
	DIPN: 19							
9	Periareolar: 50	TT: 531 NTT: 223 Perioperative use: NR	TT: 33 (3.5) NTT: 8 (2.9)	16(1.7) *	2 (0.2) *	20 (2.1) *	Surgical intervention: 27 (2.8) *	26.4 ± 16.8 months
	Buttonhole: 37							
	Keyhole: 25							
	DIFNG: 836							
10	Periareolar: 37	TT-C: 64 TT-S: 172 (Suspended 2 weeks preop)	TT-S: 13 (7.6) TT-C: 4 (6.3)	TT-S: 5 (2.9) TT-C: 0 (0)	TT-S: 5 (2.9) TT-C: 1 (1.6)	TT-S: 3 (1.7) TT-C: 0	VTE: 0	3 months
	DIFNG: 184							
	DIPN: 15							
11	Periareolar: 13	TT: 18 NTT: 26 Perioperative use: NR	5 (3.6) *	1 (0.7) *	NR	4 (2.9) *	Hypertrophic scar: 4 (2.9) *	25 months
	Keyhole: 17							
	DIFNG: 177							
	DIPN: 2							
12	NR	TT-S: 175 (Suspended 2 weeks preoperatively) TT-C: 211 NTT: 104	NTT: 3 (2.9) TT-S: 5 (2.9) TT-C: 6 (2.8)	NTT: 1 (1.0) TT-S: 2 (1.1) TT-C: 0	Partial NTT: 0 TT-S: 2 (1.1) TT-C: 1 (0.5) Complete NTT/TT-S/TT-C: 0	NTT: 2 (1.9) TT-S: 2 (1.1) TT-C: 0	VTE NTT/TT-S: 0 TT-C: 1 (0.5)	12 months
13	Periareolar: 21	TT-S: 101 (Suspended 3 weeks preoperatively) NTT: 69	TT-S: 7 (7.0) NTT: 2 (3.0)	TT-S: 4 (3.9) NTT: 5 (7.2)	TT-S: 2 (1.9) NTT: 1 (1.4)	TT-S: 1 (1.4) NTT: 1 (1.4)	Surgical intervention NTT: 10 (14.5), TT: 12 (11.9)	NR
	DIFNG: 86							
	DIPN: 53							
	Omega-shaped resection: 4							
14	Periareolar: 21	TT-S: 87 (Suspended 2 weeks preoperatively) NTT: 17	TT-S: 14 (16.1) NTT: 0	1 (1.0) *	12 (11.6)*	1 (0.9) *	NAC hypochromia 18 (17.3) *	4 months
	DIPN: 61							
	DIFNG: 21							
15	Periareolar: 8	TT: 22 Perioperative use: NR	TT: 9 breasts (40.9)	TT: 1 (2.3)	TT: 0	TT: 1 (2.3)	NR	NR
	DIPN: 2							
	DIFNG: 12							

*NR* not reported, *TT* testosterone treatment group, *NTT* non-testosterone treatment group, *TT-C* testosterone treatment continued, *TT-S* testosterone treatment stopped, *NAC* nipple areolar complex, *DIFNG* double incision with free nipple graft, *DIPN* double incision with pedicled nipple. Age and BMI are reported in mean ± SD, others are indicated in number (%) where indicated, nicotine use indicates current/recent smokers

^*^Indicates the complications could not be separated into either group or were excluded from analysis

### Preoperative Hormone Therapy and Complications

**Fig. 2:**
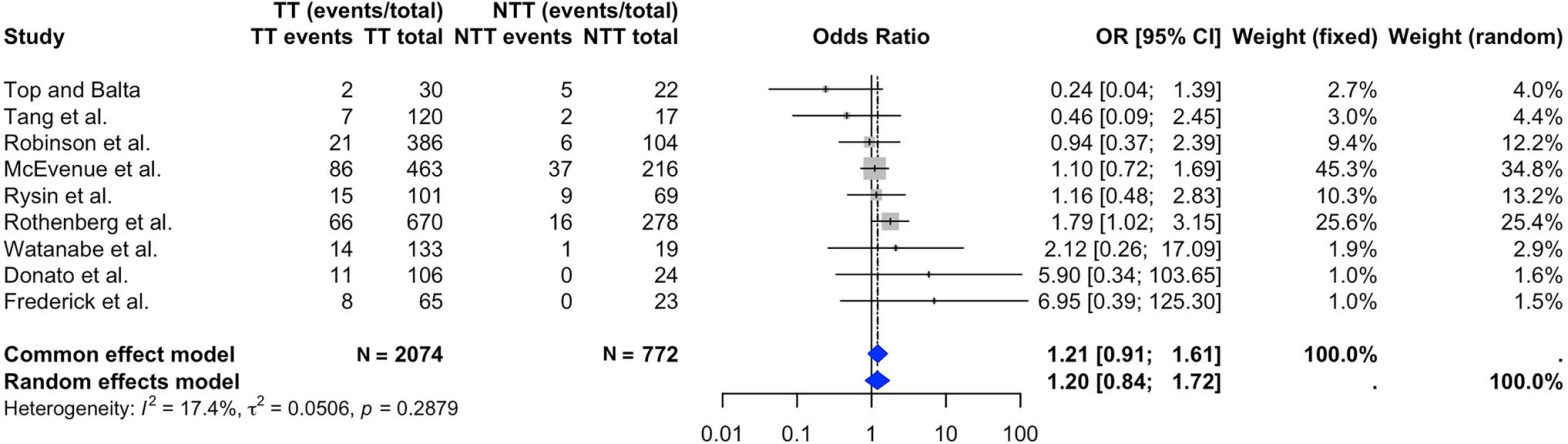
Forest plot of the meta-analysis comparing the prevalence of all acute complications between the testosterone treatment (TT) group and the non-testosterone treatment (NTT) group. The odds ratio (OR) and 95% confidence interval (CI) are shown for each study. Horizontal lines indicate the 95% CI, and the size of the square reflects the weight of each study in the meta-analysis. Acute complications included hematoma, wound infection, seroma, and nipple–areolar complex (NAC) necrosis. Heterogeneity: I^2^ = 17.4%, τ^2^ = 0.0506, Cochran’s Q test *p* = 0.2879

**Fig. 3:**
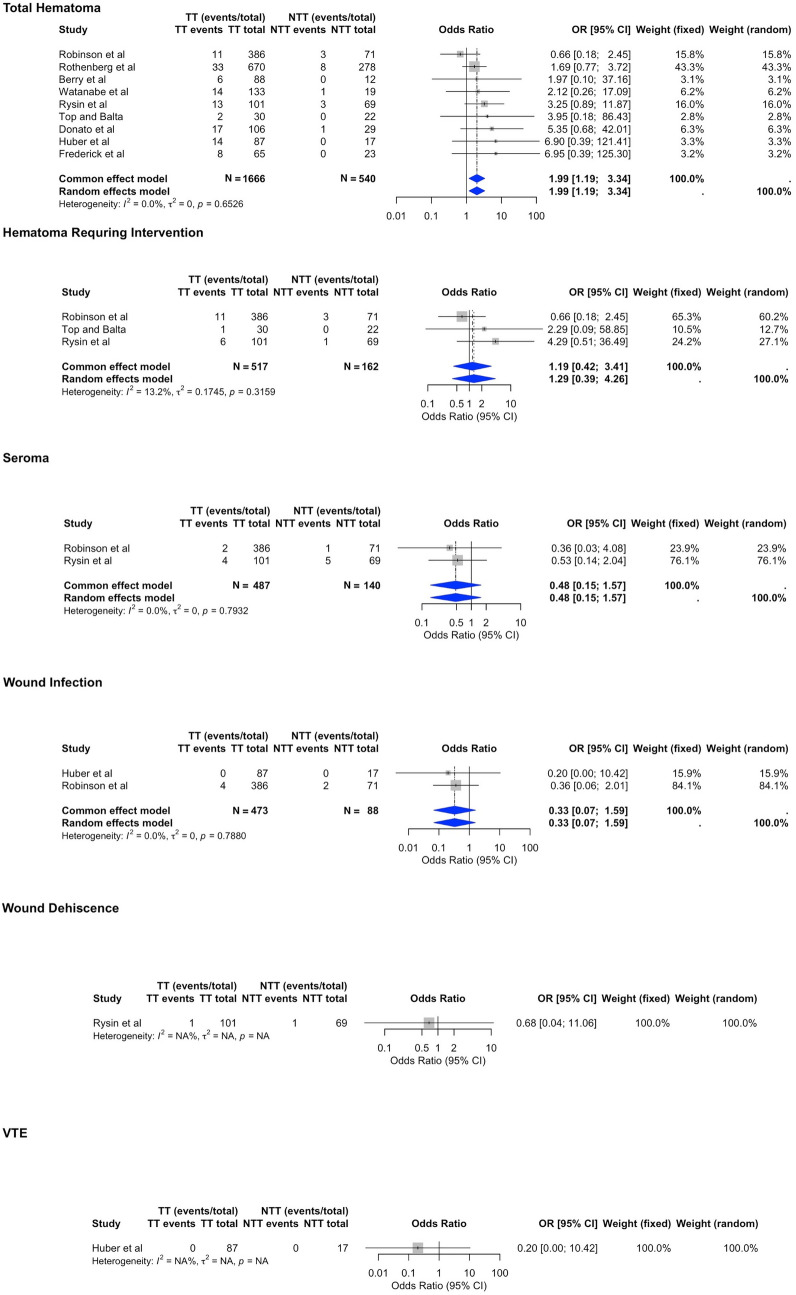
Forest plot of a meta-analysis comparing the prevalence of total hematoma, hematoma requiring intervention, seroma, wound infection, wound dehiscence, and venous thromboembolism (VTE) between the testosterone therapy (TT) and no testosterone therapy group (NTT). Each study’s odds ratio (OR) and 95% confidence interval (CI) are shown. Horizontal lines indicate the 95% CI, and the size of the square reflects the weight of each study in the meta-analysis

**Fig. 4:**
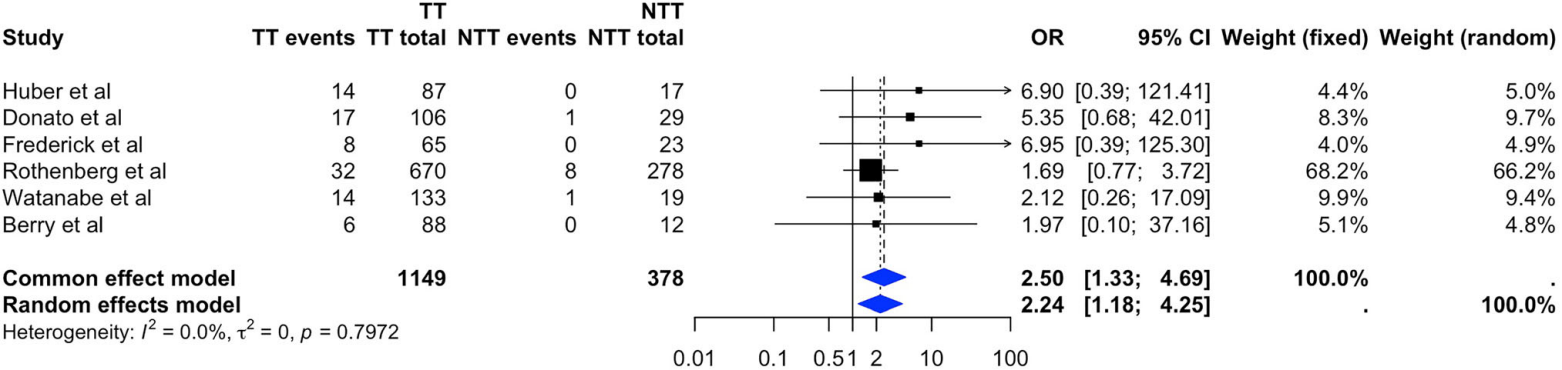
Forest plot of a sensitivity analysis excluding studies that reported nicotine use, demonstrating a stable pooled odds ratio for the outcome in patients receiving testosterone therapy (TT) compared with no testosterone therapy (NTT), with negligible heterogeneity

The overall rate of acute complications was 11.08% in the TT group and 9.80% in the NTT group (*p* = 0.29). The odds of experiencing acute complications were not significantly higher in the TT group compared to the NTT group (OR: 1.20, 95% CI: 0.84–1.72; Fig. 2). A meta-analysis of studies comparing TT with NTT demonstrated a significantly higher rate of total hematoma among TT patients (OR = 1.99 [95% CI: 1.19–3.34]), with no significant heterogeneity across studies (I^2^ = 0.0%, *p* = 0.65) (Fig. 3). In sensitivity analyses excluding studies reporting nicotine use in both groups, TT remained associated with significantly higher odds of total hematoma (random-effects OR 2.24, 95% CI 1.18–4.25; I^2^ = 0%) (Fig. 4). For hematoma requiring intervention, the pooled OR was 1.29 [95% CI: 0.39–4.26], showing no statistically significant difference between groups (I^2^ = 13.2%, *p* = 0.32).

No significant differences were observed for seroma (OR = 0.48 [95% CI: 0.15–1.57], I^2^ = 0.0%), wound infection (OR = 0.33 [95% CI: 0.07–1.59], I^2^ = 0.0%), wound dehiscence (OR = 0.68 [95% CI: 0.04–11.06]), or VTE (OR = 0.20 [95% CI: 0.00–10.42]) (Fig. 3).

Overall, testosterone therapy was significantly associated with increased odds of total hematoma, but not with other postoperative complications, and all analyses demonstrated low heterogeneity across studies.

### Perioperative Hormone Cessation and Hematoma Risk

One thousand one hundred eighty patients were included in the TT-C group, and 990 patients in the TT-S group. The average age was 21.86 ± 8.84 years in the TT-C group and 24.52 ± 8.27 years in the TT-S group. 9.00% in the TT-C group and 12.00% in TT-S group were current or recent smokers (*p* =0.34). Using pooled analysis using aggregated patient-level data, total hematoma rates were not significantly different between the TT-C and TT-S groups (8.73% vs. 7.80%; *p* = 0.54), nor were rates of hematomas requiring intervention (6.64% vs. 6.48%; *p* = 0.93). Seroma rates (0.44% vs. 2.46%) were significantly higher in the cessation group compared to the continuation group (*p* = 0.03). The risk of venous thromboembolism (0.22% vs. 0%; *p* = 0.66) was comparable between the two groups.

Table 2 depicts patient characteristics and complications between TT-C and TT-S.

**Table 2:** Table summarizing the demographics, and the prevalence of complications, between the testosterone treatment continued (TT-C) group and the testosterone treatment stopped (TT-S) group

	TT-C (%)	TT-S (%)	*p*-value
Age	21.86 ± 8.84	24.52 ± 8.27	**<0.01**
BMI	26.55 ± 5.62	27.63 ± 6.57	**0.01**
Current/Recent smokers patients (% total)	19/211 (9.00%)	21/175 (12.00%)	0.34
Total hematoma prevalence patients (% total)	49/561 (8.73%)	61/767 (7.80%)	0.54
Hematoma requiring intervention prevalence patients (% total)	33/497 (6.64%)	32/478 (6.48%)	0.93
Seroma prevalence patients (% total)	2/455 (0.44%)	11/448 (2.46%)	**0.03**
VTE prevalence patients (% total)	1/455 (0.22%)	0/87 (0%)	0.66

Bold values indicate statistical significant differences

*VTE* venous thromboembolism, *NAC* nipple areolar complex

Age and BMI are presented as mean ± standard deviation (SD). The remaining variables are reported as the number of events per sample population, followed by the corresponding percentage (%). The comparison of complications between the TT-C and TT-S groups was performed using the Fischer’s exact and chi-square test, and the corresponding p-values are reported

### Timing of Hormone Cessation and Hematoma Risk

Among the studies that stratified data based on testosterone cessation intervals, the most frequently reported interval was 2 weeks before surgery, in three studies (n =434). Other cessation intervals included 3 weeks (n = 101), 4 weeks (n = 232), and 6 weeks (n = 202). The associated hematoma rates for 2 weeks, 3 weeks, 4 weeks, and 6 weeks were 7.37%, 6.93%, 9.48%, and 9.90%, respectively (Table 3). These differences and trends were not statistically significant (*p* = 0.60, and *p* for trend = 0.21).

**Table 3:** Table summarizing the relationship between perioperative testosterone cessation duration before surgery (in weeks) and the hematoma rate

Cessation weeks	Total patients in each group	Hematoma rate
2 weeks	434	7.37%
3 weeks	101	6.93%
4 weeks	232	9.48%
6 weeks	202	9.90%

The total number of patients in each cessation group is shown alongside the corresponding hematoma rate (%). P for trend across cessation intervals = 0.21 (Cochran–Armitage test)

### Surgical Characteristics and Outcomes

Twelve studies contributed effect size estimates for total hematoma, of which only four reported incision type (limited vs. extended). In meta-regression, the model showed no residual heterogeneity (τ^2^ = 0.00, I^2^ = 0%). Incision type was not a significant moderator of the association between testosterone therapy (TT) and hematoma risk compared with no testosterone therapy (NTT) (QM (1) = 0.34, *p* = 0.56). The regression slope (log OR = 1.10, 95% CI −2.57 to 4.76) indicated no meaningful relationship between incision type and the odds of hematoma in TT versus NTT.

On pooled analysis using aggregated patient-level data, which assessed the main effect of incision type without stratification by testosterone status, total hematomas were observed in 6.52% (80 out of 1,227) of patients undergoing extended incision mastectomy, compared to 11.96% (56 out of 468) of patients undergoing the limited incision technique (*p* < 0.01), independent of hormone status.

In the TT group, 14.26% of patients underwent double incision with a pedicled nipple areola, and 61.75% underwent double incision with a free nipple areola graft, compared to 6.93% and 76.56% in the NTT group, respectively (*p* < 0.01 for both comparisons). When hormone therapy was not considered, total NAC necrosis rates were 0.38% in pedicled NAC procedures and 0.47% in free nipple areola graft procedures (*p* = 0.88). Partial NAC necrosis occurred in 4.29% of pedicled NAC procedures compared to 1.5% in free NAC graft procedures, showing a statistically significant difference (*p* = 0.02). Pooled analysis for NAC necrosis based on testosterone use was not feasible because of data limitations.

### Bias Assessment

**Fig. 5:**
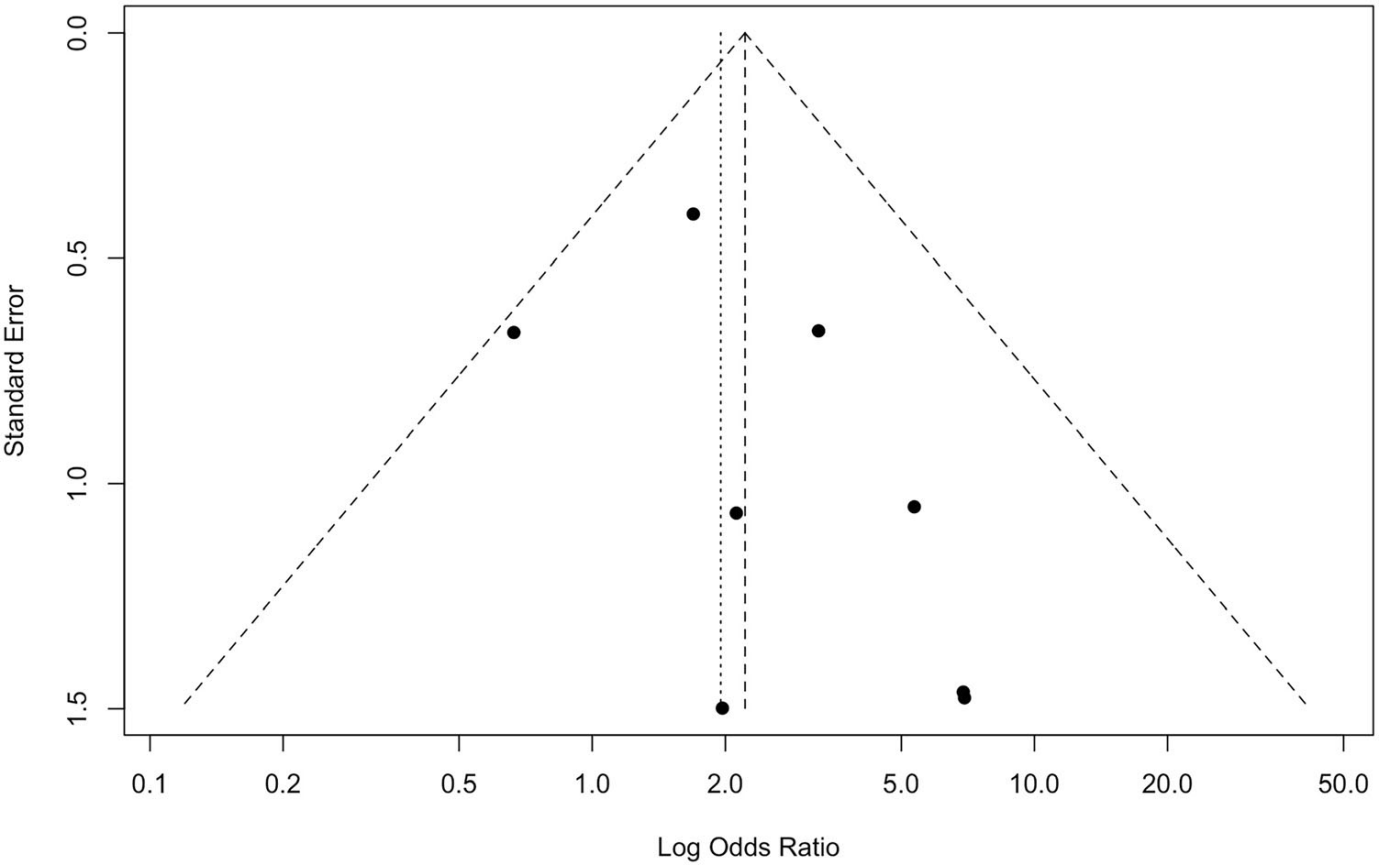
Funnel plot assessing publication bias for hematoma rates between testosterone therapy (TT) and no testosterone therapy (NTT). Study-specific log odds ratios are plotted against their standard errors. Visual inspection demonstrates no marked asymmetry

Visual inspection of the funnel plot for total hematoma did not demonstrate marked asymmetry, suggesting no strong evidence of publication bias (Fig. 5). Formal statistical testing for small-study effects was not performed due to the limited number of studies.

## Discussion

Although GAHT, particularly testosterone, has been shown to significantly improve the well-being and psychosocial functioning of transgender individuals, it is also reported to be associated with increased surgical complications, such as delayed wound healing and wound dehiscence, in gender-affirming surgeries [16–20]. While some centers advocate for suspending testosterone in the perioperative period, others continue therapy, leading to inconsistent practices across institutions. This variation highlights the need to better understand how testosterone influences post-operative recovery and short-term complications, to ensure optimal care for transgender individuals undergoing surgery.

The overall rate of hematoma was found to be significantly higher in patients receiving testosterone supplementation compared to testosterone naïve. Most studies directly comparing complication rates between TT and NTT groups observed a trend toward higher hematoma rates in the TT group, though these trends were not always statistically significant [8, 13, 15, 16]. By performing a meta-analysis, we increased the sample size, improving the reliability of findings and addressing limitations of individual studies with smaller cohorts. Basic science studies have shown that androgens, via androgen receptor-mediated upregulation of vascular endothelial growth factor (VEGF) and endothelial proliferation, promote neovascularization and capillary density [17–21]. In the surgical setting, this heightened vascularity may lead to greater intraoperative bleeding and increased susceptibility to postoperative hematoma, particularly when combined with limited exposure or difficulty in achieving meticulous hemostasis. Previous studies have reported that limited incision mastectomy carries a significantly higher risk of hematoma compared with extended or double-incision mastectomy [22]. Differences in hematoma rates between extended and limited incision techniques are likely related to technical factors, with limited access increasing the potential for vessel injury and obscuring visualization of bleeding points [22]. In our pooled analysis using aggregated patient-level data, when data were combined without accounting for hormone status, we similarly observed higher hematoma rates in the limited incision group compared with extended incision mastectomy. However, meta-regression of hematoma rates between TT and NTT patients, controlling for surgical approach (extended vs. limited incision), revealed no significant effect of incision type. This suggests that hormone status is independently associated with hematoma risk, irrespective of incision technique. Nonetheless, given the small number of studies contributing incision type data (k = 4), these results should be interpreted as exploratory and hypothesis-generating rather than confirmatory.

Our analysis found no significant difference in overall complication rates between patients who continued hormone therapy and those who stopped it. The VTE rate in both groups (TT and NTT) was comparable to that reported in cosmetic plastic surgery patients (0% and 0.14% vs. 0.20%) [23]. Full-thickness necrosis of the NAC in both pedicled and free nipple graft techniques was low and showed no significant difference between the two approaches [24]. However, we observed significantly higher rates of partial-thickness NAC necrosis in pedicled NAC compared to free NAC grafts (4.29% vs. 1.5%; *p* = 0.015), independent of hormone therapy use. This interesting difference might be related to extreme thinning of the NAC pedicle to avoid chest fullness in pedicled techniques and could be used to counsel the patients.

Further subgroup analysis of testosterone supplementation in our study revealed no significant differences in overall complication rates, including hematoma rates, or VTE incidence, between TT-C and TT-S. This finding aligns with prior studies suggesting that the continuation of testosterone during the perioperative period does not significantly increase surgical complications in gender affirming mastectomy [25]. The rate of seroma formation was significantly higher among patients who discontinued testosterone compared to those who continued perioperatively (2.46% vs. 0.44%). No established biologic mechanism directly links testosterone cessation to seroma formation. Overall seroma event rates were within the ranges reported for breast surgery [26]. Although the testosterone cessation group was modestly older (24.52 ± 8.27 vs 21.86 ± 8.84) and had a higher mean BMI (27.63 ± 6.57 vs 26.55 ± 5.62), both reported risk factors for seroma formation; it is unclear whether these small differences alone account for the observed variation in seroma rates [27–30]. The difference may also have been influenced by perioperative factors not consistently reported across studies, including use of electrocautery for dissection, timing of drain removal, drain suction characteristics, obliteration of dead space, and postoperative shoulder mobilization protocols [31].

Therefore, considering that abrupt cessation of hormone therapy can have adverse psychological and physiological consequences, including worsened gender incongruence, mood disturbances, and decreased energy levels, which are particularly concerning for transgender individuals, continuation of testosterone supplementation perioperatively appears to be a viable option [9, 32].

### Limitations

The primary limitation of this systematic review is the quality and completeness of available data, particularly in the stratification of results based on hormone status and surgical characteristics. Although patients were classified as TT, NTT, TT-C, or TT-S based on reported hormone status, details such as dosing regimen, route of administration, treatment adherence, and laboratory confirmation of physiologic effect were inconsistently reported. Minimum treatment duration and precise exposure windows were not consistently documented, limiting standardization of these parameters. This lack of standardization introduces the potential for exposure misclassification, which may partly explain why perioperative testosterone cessation did not appear to significantly influence hematoma rates. Variability in surgical technique, surgeon experience (which was inconsistently reported and could not be quantitatively analyzed), and institutional practices may also influence reported complication rates. Definitions of hematoma and thresholds for intervention may have varied across studies, which may have contributed to outcome heterogeneity and limited direct comparison of severity across cohorts.

Outcomes were stratified based on surgical approach, and a meta-regression was conducted to evaluate the moderating effect of technique; however, these analyses are limited by inconsistent reporting and a lack of standardized data on surgeon experience and perioperative management. Although comorbidities such as hypertension, diabetes, nicotine use, and coagulation disorders were collected when reported, these data were inconsistently available and variably defined across studies. Study-level sensitivity analyses excluding studies reporting nicotine use reduced between-group differences in smoking prevalence; however, residual confounding by nicotine use remains possible due to the lack of patient-level data.

Assessment of publication bias was limited by the small number of included studies. Patients receiving testosterone therapy may differ systematically from non-testosterone users, introducing potential selection bias. Furthermore, information regarding perioperative anticoagulation or antiplatelet medication use was rarely provided. This likely reflects the younger and generally healthy demographic undergoing gender-affirming mastectomy; however, due to data limitations, it precluded a detailed analysis of potential confounding effects related to these variables. All these limit the strength of the conclusions that can be drawn.

## Conclusions

Preoperative testosterone therapy appears to be associated with higher rates of total hematoma following gender-affirming mastectomy compared to those who had never used testosterone. However, discontinuing testosterone in patients already on therapy did not significantly affect complications or hematoma rates. Decisions regarding the timing of hormone therapy initiation should be carefully weighed against its established mental health benefits. Overall, these findings support heightened perioperative awareness and vigilance for hematoma risk in patients receiving testosterone. Nonetheless, these results should be interpreted with caution, as the available evidence is primarily clinical and lacks corroborating basic science data to fully elucidate the underlying mechanisms. Future prospective studies with standardized outcome definitions and detailed reporting of hormone exposure, surgical variables, and perioperative factors are needed to better clarify risk and underlying mechanisms.
